# Airbrush-based solution blow spinning of cellulose acetate/poly(vinyl acetate) blends for nonwoven mat formation

**DOI:** 10.1039/d6ra03209e

**Published:** 2026-10-03

**Authors:** Maria Emad Bashawri, Abimbola O. Orisawayi, Sameer S. Rahatekar, Krzysztof K. Koziol

**Affiliations:** a Centre for Materials, Faculty of Engineering and Applied Sciences, Cranfield University Cranfield Bedfordshire MK43 0AL UK maria.bashawri@cranfield.ac.uk k.koziol@cranfield.ac.uk; b School of Mechanical Electrical & Manufacturing Engineering, Loughborough University Loughborough Leicestershire LE11 3TU UK S.S.Rahatekar@lboro.ac.uk

## Abstract

The global textile industry faces increasing pressure to adopt cleaner and more resource-efficient manufacturing technologies. Solution blow spinning (SBS) has emerged as a promising route for nonwoven production, offering rapid fibre deposition, simple equipment, and direct *in situ* formation on substrates. In this study, an airbrush-based SBS process was used to fabricate spray-formed nonwoven structures from an epoxy-free cellulose acetate (CA)/poly(vinyl acetate) (PVAc) formulation. Particular emphasis was placed on understanding the concentration-dependent transition from droplet-dominated deposition to continuous fibre formation. Formulation screening revealed a narrow processability window, with polymer concentration identified as the critical parameter governing jet stability, fibre morphology, and nonwoven cohesion. A clear transition from droplet-rich deposition to stable fibre-forming behaviour was observed as polymer concentration increased, with cohesive, peelable nonwoven mats successfully produced at 23 wt%. Scanning electron microscopy analysis revealed the formation of a continuous interconnected fibrous network with an average fibre diameter of 0.97 ± 0.41 µm. Fourier transform infrared spectroscopy confirmed preservation of the chemical integrity of both polymer components after spraying, while thermogravimetric and differential scanning calorimetry analyses demonstrated adequate thermal stability and predominantly amorphous behaviour. The resulting nonwoven exhibited an average thickness of 0.595 ± 0.049 mm, porosity of 66.0 ± 3.5%, and preliminary mechanical integrity as a self-supporting fibrous structure. These findings provide mechanistic insight into spray-based fibre formation and demonstrate that airbrush-based SBS offers a simple, low-cost, and scalable route for producing cohesive fibrous nonwoven structures, expanding the application potential of SBS beyond conventional biomedical and filtration systems.

## Abstract 2

Airbrush solution blow spinning enabled direct fabrication of cohesive CA/PVAc nonwoven mats. Optimising polymer concentration shifted deposition from droplets to continuous fibres, yielding lightweight, porous, textile-like structures.

## Graphical abstract

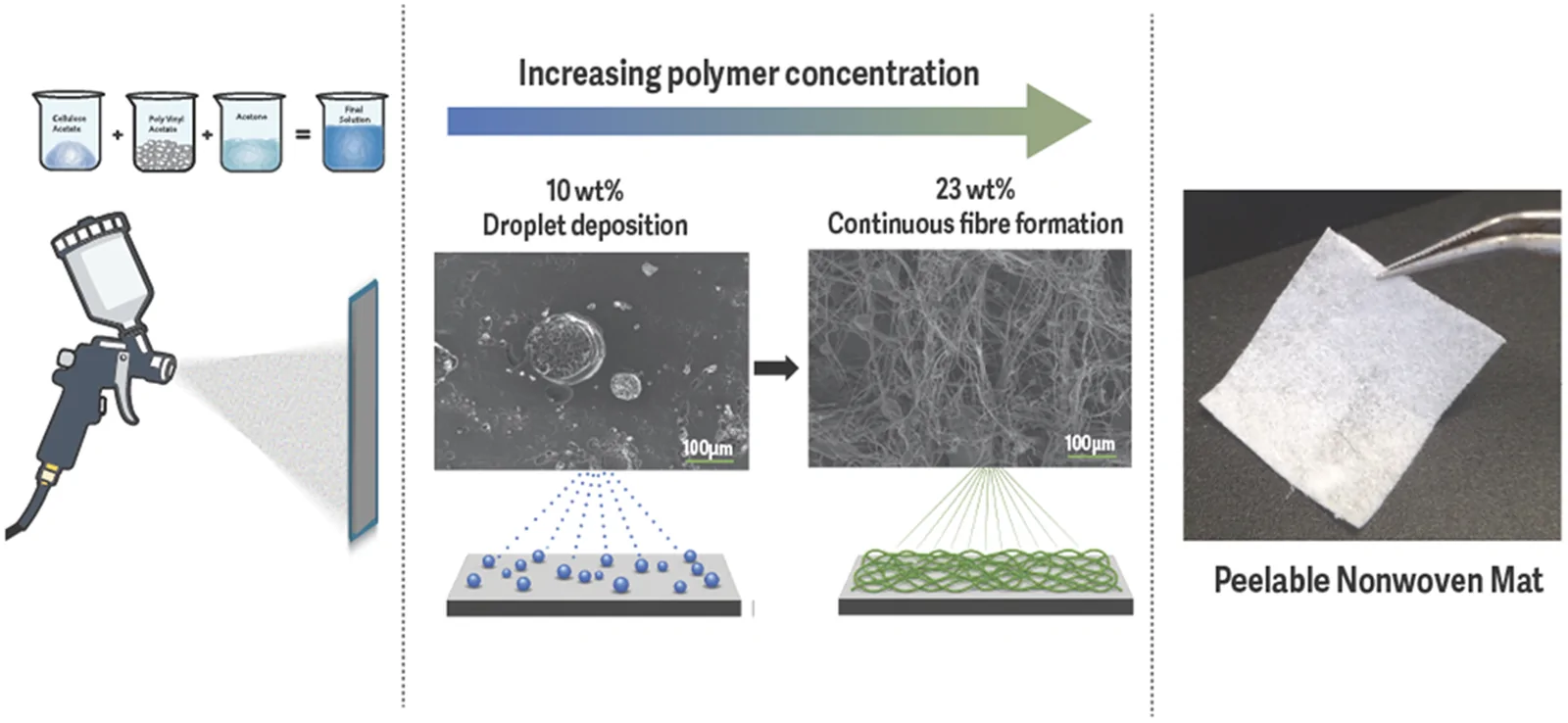

## 1 Introduction

The textile industry is one of the most resource intensive sectors globally, owing to its high consumption of water, energy, and chemicals and substantial contribution to environmental degradation.^1,2^ In response to increasing sustainability pressures, the industry has begun shifting toward cleaner and more resource-efficient textile manufacturing technologies. Among emerging alternatives, nonwoven fabrication offers a promising route for direct textile formation from fibres or polymer streams by eliminating energy-intensive yarn spinning and weaving processes, thereby reducing both processing complexity and environmental footprint.^3,4^ Despite these advantages, most commercial nonwovens continue to rely on petroleum derived polymers such as polypropylene and polyester, which limits their biodegradability and end of life sustainability.^5^

Consequently, growing attention has turned to bio-based and biodegradable nonwoven alternatives which are manufactured from renewable feedstocks such as cellulose, polylactic acid (PLA), and starch-based polymers.^6–8^ Among polymer-laid nonwoven manufacturing techniques, electrospinning and solution blow spinning (SBS) have attracted considerable interest due to their ability to generate continuous micro and nanofibers directly from polymer solutions.^7^ Electrospinning has been widely studied because of its ability to produce fibres with controlled morphology, alignment, and high surface area.^9^ However, despite its effectiveness at the laboratory scale, electrospinning faces significant barriers to industrial adoption, requiring high voltage operation, low production rates, and operational safety concerns that limit large scale or industrial implementation.^7^ Furthermore, electrospinning systems rely on volatile organic solvents and non-renewable polymers,^10^ introducing additional environmental challenges.

To overcome these challenges, solution Blow Spinning has been proposed as a promising alternative, as it employs pressured air instead of an electric field through a small nozzle to elongate the polymer solution into fibres as the solvent evaporates. SBS offers several practical advantages, including higher production rates, simpler and safer operation, reduced equipment complexity, and the capability for direct *in situ* fibre deposition onto target substrates.^11–14^ Nevertheless, the majority of SBS studies to date have focused on submicron fibre generation and porous nonwoven mats for biomedical, environmental, and protective applications such as wound dressings, tissue scaffolds, and filtration media.^14–17^ Comparatively little attention has been given to the formation of cohesive fibrous nonwoven structures with sufficient mechanical integrity for handling, peeling, or textile-related applications. More importantly, the concentration-dependent mechanism governing the transition from droplet deposition to continuous fibre formation, particularly the role of polymer chain entanglement in stabilising the spray jet, remains poorly understood in spray-based nonwoven textile systems.

Earlier demonstrations of spray formed textiles known as Fabrican, developed by Torres and Luckham, established the feasibility of producing wearable spray-on fabrics directly from polymer-based formulations.^18^ Although this system demonstrated the practical potential of spray-applied textile formation, cohesion within the deposited material relied heavily on epoxy resins or synthetic adhesive binders. Such chemically intensive components may introduce concerns related to environmental burden, limited biodegradability, and potential dermal irritation.^19^ Consequently, the development of binder-efficient or epoxy-free sprayable systems based on biodegradable polymers remains an important unresolved challenge for sustainable nonwoven fabrication.

In this context, bio-derived and biodegradable polymers such as cellulose derivatives, poly (vinyl alcohol), and polylactic acid have emerged as particularly promising alternatives to fossil-based polymers used in conventional nonwoven technologies.^20–23^ Among these, cellulose acetate (CA) offers advantages due to its biodegradability, film-forming ability, and potential to provide structural integrity during fibre deposition. Acetone was selected as the solvent because its high volatility and low boiling point promote rapid solvent evaporation, which is beneficial for fibre solidification during solution blow spinning.^24^ However, neat cellulose acetate has been reported to exhibit limited spinnability under SBS conditions, often producing bead-rich morphologies and suffering from nozzle clogging due to rapid solvent evaporation, thereby limiting the formation of uniform fibrous structures.^25^ To overcome this limitation, poly(vinyl acetate) (PVAc) was incorporated as a secondary polymer to enhance solution viscosity and polymer chain entanglement, enabling stable solution blow spinning and the production of continuous nonwoven fibrous structures.^26^

In this study, an epoxy-free cellulose acetate/poly(vinyl acetate) (CA/PVAc) formulation was developed for airbrush-based solution blow spinning to produce cohesive spray-formed nonwoven structures. The concentration-dependent transition from droplet-dominated deposition to continuous fibre formation was investigated, with polymer concentration identified as a key parameter governing jet stability, fibre morphology, and nonwoven cohesion. A comparative analysis between low (10 wt%) and fibre-forming (23 wt%) polymer concentrations, supported by intermediate formulation screening, was conducted to investigate this droplet-to-fibre transition mechanism. The resulting nonwoven structures were characterised using scanning electron microscopy (SEM), Fourier transform infrared spectroscopy (FTIR), thermogravimetric analysis (TGA), differential scanning calorimetry (DSC), and preliminary physical and mechanical testing. This work provides mechanistic insight into spray-based nonwoven formation and establishes a scalable, low-cost platform for producing cohesive, peelable, textile-related nonwoven structures, thereby suggesting broader application potential for solution blow spinning beyond conventional biomedical and filtration systems. Such structures may be particularly relevant for applications requiring lightweight, conformable, and removable fibrous layers, including disposable textile materials, protective coverings, and other on-demand spray-deposited nonwoven systems.

## 2 Materials and methods

### 2.1 Materials

Cellulose acetate (CA, *M*_w_ ≈ 100 000 g mol^−1^, CAS 9004-35-7) and acetone (technical grade, *M*_w_ = 58.08 g mol^−1^, CAS 67-64-1) were purchased from Fisher Scientific (Loughborough, UK). Poly(vinyl acetate) (PVAc, *M*_w_ ≈ 100 000 g mol^−1^, bead form, CAS 9003-20-7) was purchased from Sigma-Aldrich (UK). All materials were used as received without further purification.

### 2.2 Methods

#### 2.2.1 Preparation of CA/PVAc polymer solutions

Preliminary formulation screening was conducted to establish the processability window of the CA/PVAc system under airbrush-based solution blow spinning (SBS) conditions. Multiple formulations with varying total polymer concentrations (10–23 wt%) and selected CA/PVAc ratios were prepared and evaluated based on sprayability, nozzle behaviour, jet stability, and deposition characteristics. Following this optimisation stage, representative low- and high-concentration formulations were selected for detailed structural, thermal, and mechanical characterisation.

All formulations were prepared with a total batch mass of approximately 50 g. For the low-concentration formulation, poly(vinyl acetate) (PVAc, 7 wt%) was first dissolved in acetone (90 wt%) at ambient temperature (≈25 °C) under continuous magnetic stirring for 2 h. Cellulose acetate (CA, 3 wt%) was then added and stirred for an additional 1 h until a visually homogeneous solution was obtained, yielding a polymer solution with a total concentration of 10 wt%.

Using the same preparation procedure and an approximately 50 g total batch mass, a second formulation with a higher polymer concentration (23 wt%) was prepared by increasing the CA and PVAc contents to 9.8 wt% and 13 wt%, respectively. All solutions were used immediately after preparation to minimise solvent evaporation and viscosity changes prior to spraying. This preparation protocol followed a previously reported mixing strategy for stable acetone-based polymer systems.^27^ The 10 wt% and 23 wt% formulations were selected as representative low- and high-concentration systems corresponding to the two extreme processing regimes identified during formulation optimisation, namely droplet-dominated deposition and stable fibre-forming deposition, respectively.

#### 2.2.2 Airbrush-based solution blow spinning deposition

Nonwoven mats were produced by solution blow spinning (SBS) using a ROIIKETU BL009 high volume low pressure (HVLP) mini spray gun equipped with a 0.8 mm stainless-steel single-fluid nozzle and a 125 mL gravity feed cup (ROIIKETU, UK), connected to a compressed air supply. The operating air pressure was maintained at 4 bar (≈58 psi) throughout all experiments.

During deposition, the polymer solutions were sprayed onto a flat low-density polyethylene (LDPE) collector. To account for concentration-dependent differences in solution viscosity and spray behaviour, the working distance between the nozzle and collector was adjusted to 25 cm for the 10 wt% formulation and 15 cm for the 23 wt% formulation. Intermediate formulations (15–18 wt%) were deposited using the same working distance as the 23 wt% formulation (15 cm).

To primarily evaluate the effect of polymer concentration on fibre formation, all major processing parameters were kept constant, including nozzle geometry, operating pressure, solvent system, spraying motion, and deposition strategy. Spray distance was adjusted only when necessary to accommodate formulation-dependent deposition behaviour.

Deposition was performed using a manual horizontal side-to-side spraying motion to promote uniform fibre distribution across the collector surface. For multilayer nonwoven fabrication, four successive spray layers were deposited with approximately 2 min drying intervals between layers to allow partial solvent evaporation and progressive fibre accumulation. A schematic illustration of the solution preparation, airbrush deposition setup, and nonwoven collection procedure is shown in Fig. 1.

**Fig. 1:**
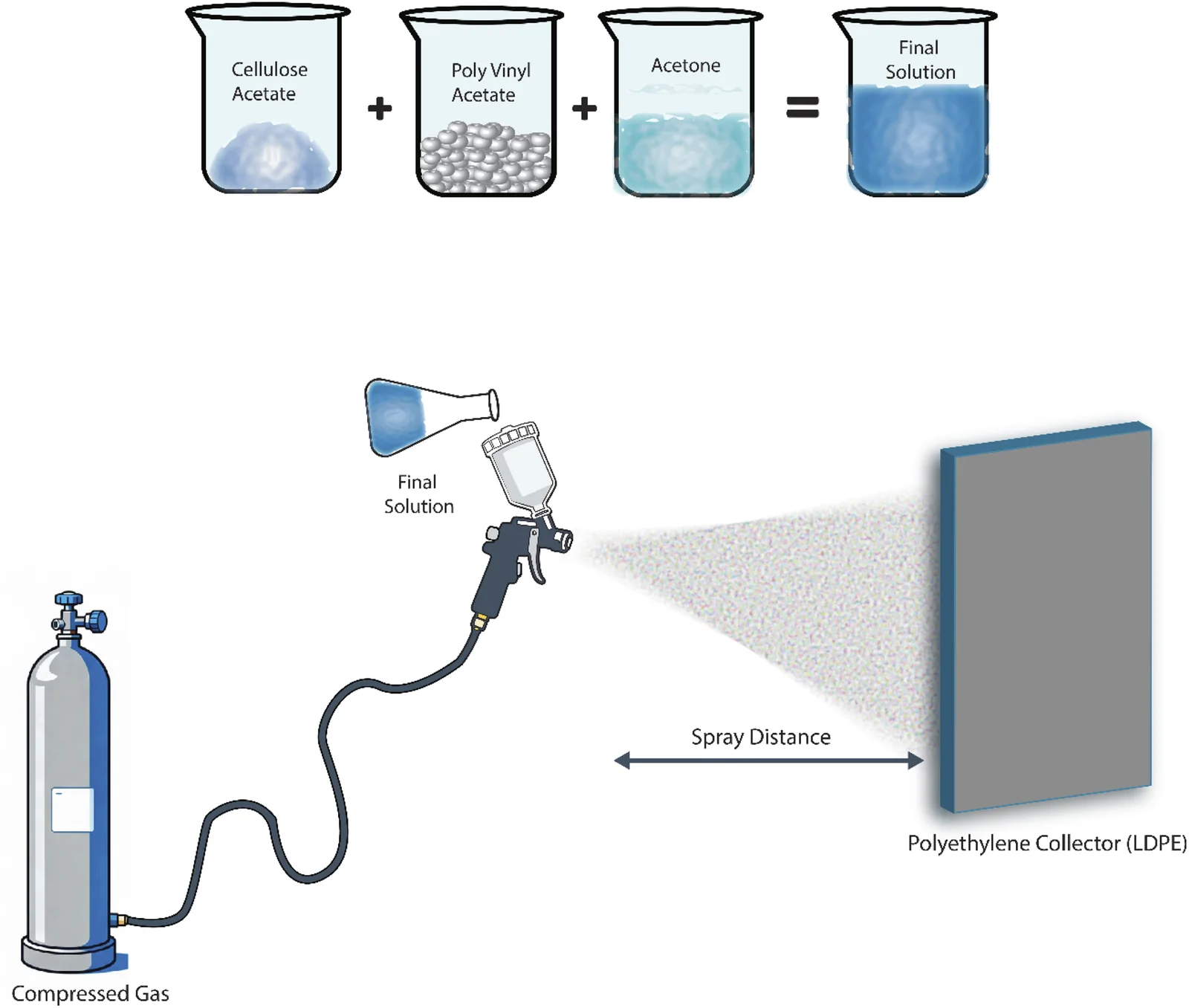
Schematic illustration of the airbrush-based solution blow spinning (SBS) setup used for fabrication of CA/PVAc nonwoven mats, showing polymer solution preparation, spray deposition, and fibre collection on the LDPE collector.

#### 2.2.3 Characterisation methods

##### 2.2.3.1 Optical microscopy

The surface morphology and deposition uniformity of representative low- and high-concentration samples (10 wt% and 23 wt%) were examined using an optical microscope (Spectrographic BS-5062TTR, UK) equipped with a digital camera. Images were acquired at 10× magnification to assess surface coverage, fibre distribution, and deposition uniformity.

##### 2.2.3.2 Scanning electron microscopy (SEM)

The microstructural morphology of the CA/PVAc nonwoven samples was characterized using a TESCAN S8000 scanning electron microscope (TESCAN, Kohoutovice, Czech Republic). Samples were sputter-coated with gold under an argon atmosphere prior to imaging using an Emitech K250 sputter coater and imaged at an accelerating voltage of 10 kV.

Fibre diameter analysis was determined only for the fully fibrous 23 wt% sample.

Diameter distributions were determined from at least four representative SEM micrographs using ImageJ (Fiji v1.54), and a total of 100 individual fibres were measured for statistical analysis.

##### 2.2.3.3 Fourier transform infrared spectroscopy (FTIR)

Fourier transform infrared spectra were acquired with a Thermo Scientific™ Nicolet iS10 spectrometer fitted with a diamond attenuated total reflectance (ATR) crystal. Spectra were collected from 4000 to 500 cm^−1^ at a resolution of 4 cm^−1^ with 50 co-added scans at room temperature and processed using OMNIC software.

FTIR measurements were performed on neat cellulose acetate (CA), neat poly(vinyl acetate) (PVAc), and the representative sprayed CA/PVAc nonwoven mat to compare characteristic absorption bands and assess the preservation of functional groups following solution blow spinning and solvent evaporation.

##### 2.2.3.4 Thermogravimetric analysis (TGA)

Thermal stability was evaluated using a Discovery TGA 550 (TA Instruments, USA) equipped with a high-sensitivity microbalance (±0.1 µg) and automated gas delivery system. Approximately 5–10 mg of each sample (neat cellulose acetate, neat poly(vinyl acetate), and the representative sprayed CA/PVAc nonwoven mat) was placed in open platinum pans and heated from 30 to 600 °C at a heating rate of 10 °C min^−1^ under a nitrogen atmosphere with a purge flow of 50 mL min^−1^. The experimental procedure was adapted from previously reported studies.^27–29^

##### 2.2.3.5 Differential scanning calorimetry (DSC)

The thermal behaviour of the materials was studied using a Discovery DSC 250 (TA Instruments, USA) equipped with Fusion Cell™ and Tzero® technology. Samples with masses in the range of 7–10 mg (CA: 7.50 mg; PVAc: 9.70 mg; sprayed CA/PVAc blend: 9.30 mg) were sealed in Tzero® aluminium pans.

Pure cellulose acetate (CA) was analysed using a heat–cool–heat protocol to minimise thermal history effects. The sample was first equilibrated at 50 °C for 2 min, heated from 50 to 260 °C at a rate of 10 °C min^−1^ and held isothermally at 260 °C for 3 min, cooled to 50 °C at the same rate, and reheated to 260 °C.

Pure poly(vinyl acetate) (PVAc) was analysed using a two-cycle heating protocol. The sample was heated from −20 to 120 °C at 10 °C min^−1^, cooled to −20 °C, and then reheated from −20 to 200 °C at 10 °C min^−1^.

The sprayed CA/PVAc nonwoven mat was analysed using a heat–cool–heat protocol over the range −20 to 260 °C at 10 °C min^−1^. Glass transition temperatures (*T*_g_) were determined from the second heating cycle using TA TRIOS software to minimise thermal history effects.

##### 2.2.3.6 Thickness and porosity measurements

The thickness of the spray-deposited CA/PVAc nonwoven samples was measured using a digital micrometre. Measurements were taken at three different positions across each sample, and the average thickness was calculated. Five replicate samples (*n* = 5) were analysed, and the results were reported as mean ± standard deviation.

Porosity of the fibrous nonwoven samples was determined using a gravimetric method adapted from Tutak *et al.*,^30^ and calculated using the following equation:
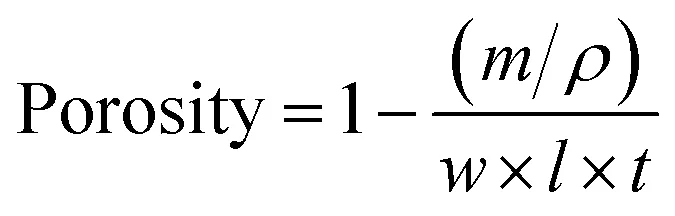
where *m* is the sample mass (g), *ρ* is the theoretical density of the CA/PVAc polymer blend (g cm^−3^), *w* is the sample width (cm), *l* is the sample length (cm), and *t* is the sample thickness (cm).

Samples were cut into 10 mm × 10 mm squares. Mass was measured using an analytical balance. The corresponding average thickness values were used in the porosity calculation. Porosity measurements were performed for five replicate samples (*n* = 5) and the values were expressed as mean ± standard deviation.

The polymer density values used for porosity estimation were obtained from supplier technical data sheets, where cellulose acetate (CA) and poly(vinyl acetate) (PVAc) densities were reported as approximately 1.27 g cm^3^ (ref. 31) and 1.18 g cm^3^,^32^ respectively. A theoretical density for the CA/PVAc blend of approximately 1.22 g cm^−3^ was estimated from the formulation composition and constituent polymer densities and was used in the porosity calculations.^33^

##### 2.2.3.7 Tensile testing

Owing to the small dimensions of the laboratory-scale SBS nonwoven samples, a modified tensile testing method based on the principles of ISO 9073-3 was employed.^34^ The specimen dimensions and gauge length were adjusted to accommodate the limitations of the testing system.

Tensile properties were measured using a DEBEN 200 N micro-tensile testing stage (Deben UK Ltd, Suffolk, UK) equipped with a 10 N load cell. Rectangular specimens measuring approximately 25 mm × 10 mm were prepared from the nonwoven sheets. The thickness of each specimen was measured individually prior to testing. The gauge length was set at 10 mm, and testing was conducted at a crosshead speed of 0.5 mm min^−1^ until specimen failure, defined as near complete loss of load bearing capacity.

Force displacement data were recorded continuously and converted to engineering stress strain curves using the measured specimen dimensions and initial gauge length. Five replicate specimens (*n* = 5) of the optimized 23 wt% polymer concentration formulation were tested, and the mean values and standard deviations were calculated.

## 3 Results and discussion

### 3.1 Effect of polymer concentration on spray processing and fibre formation

Preliminary formulation screening was conducted to establish the concentration-dependent processability window of the CA/PVAc system during airbrush-based solution blow spinning (SBS). Emphasis was placed on identifying the transition from droplet-dominated deposition to continuous fibre formation, as this transition governs the formation of cohesive textile-like nonwoven structures. Formulations with varying total polymer concentrations and CA/PVAc ratios exhibited distinct spray behaviours, ranging from dilute atomisation and nozzle clogging to stable fibre-forming deposition, as summarised in Table 1. Although both total polymer concentration and CA/PVAc ratio influenced spray behaviour, the transition toward stable fibre formation was primarily associated with increasing overall polymer concentration and the resulting enhancement in polymer chain entanglement.

**Table 1:** Summary of preliminary formulation screening, spray behaviour, and deposition morphology of CA/PVAc solutions during airbrush-based solution blow spinning (SBS)

Formulation	PVAc (wt%)	CA (wt%)	Acetone (wt%)	Total polymer (wt%)	Working distance (cm)	Spray behaviour	Deposition morphology
T1	7.0	3.0	90.0	10.0	25	Stable atomisation; highly dilute spray	Predominantly droplet-rich deposition
T2	5.5	9.5	85.0	15.0	15	Excessively viscous; nozzle clogging	Spray failure/no recoverable deposition
T3	8.5	8.7	82.8	17.2	15	Sprayable; unstable jet	Partial fibre formation with bead-like structures
T4	10.0	8.0	82.0	18.0	15	Improved sprayability	Increased fibre formation with residual beads
T5	13.0	9.8	77.0	23.0	15	Stable, reproducible deposition	Continuous interconnected fibrous nonwoven mat

At low polymer concentration (10 wt%), the solution was readily atomised but produced mainly droplet-rich deposits with poor structural integrity, preventing formation of a self-supporting sheet. This suggests that the polymer concentration was below the effective chain entanglement threshold, meaning that the polymer chains were not sufficiently interconnected to maintain a stable stretching jet during spraying. Under these dilute conditions, polymer chain interactions were insufficient to overcome capillary forces, causing the jet to break into droplets rather than form continuous fibres, consistent with behaviour reported for dilute polymer solutions.^35^

At 15 wt% total polymer concentration, the CA-rich formulation exhibited excessive viscosity and severe nozzle clogging, preventing stable spray delivery. As a result, no material was successfully deposited onto the collector for further evaluation. This behaviour indicates that polymer concentration alone did not determine processability, as the relative CA/PVAc composition also strongly influenced solution flow behaviour. The higher CA content increased solution viscosity sufficiently to impair atomisation and nozzle flow.

In contrast, intermediate formulations at 17.2–18 wt% became sprayable and exhibited partial fibre formation with residual bead-like structures. This behaviour represents a transitional regime in which polymer chain interactions were sufficient to initiate jet stretching and fibre formation but remained insufficient to fully suppress capillary-driven jet breakup.

Further increasing the polymer concentration to 23 wt% increased polymer chain overlap and entanglement density, thereby enhancing viscoelastic resistance against capillary-driven jet breakup during aerodynamic stretching. This behaviour is consistent with mechanistic models of solution blow spinning, in which fibre formation is governed by the balance between aerodynamic drag forces, viscoelastic stresses arising from polymer chain entanglement, and surface tension-driven jet breakup.^36^ When viscoelastic resistance becomes sufficiently high to counteract surface tension, sustained jet elongation can be maintained, enabling deposition of a continuous interconnected fibrous mat that could be readily removed from the collector as a cohesive nonwoven structure. As illustrated in Fig. 2, increasing polymer concentration from 10 wt% to 23 wt% resulted in a clear transition from droplet-dominated deposition to continuous fibre formation.

**Fig. 2:**
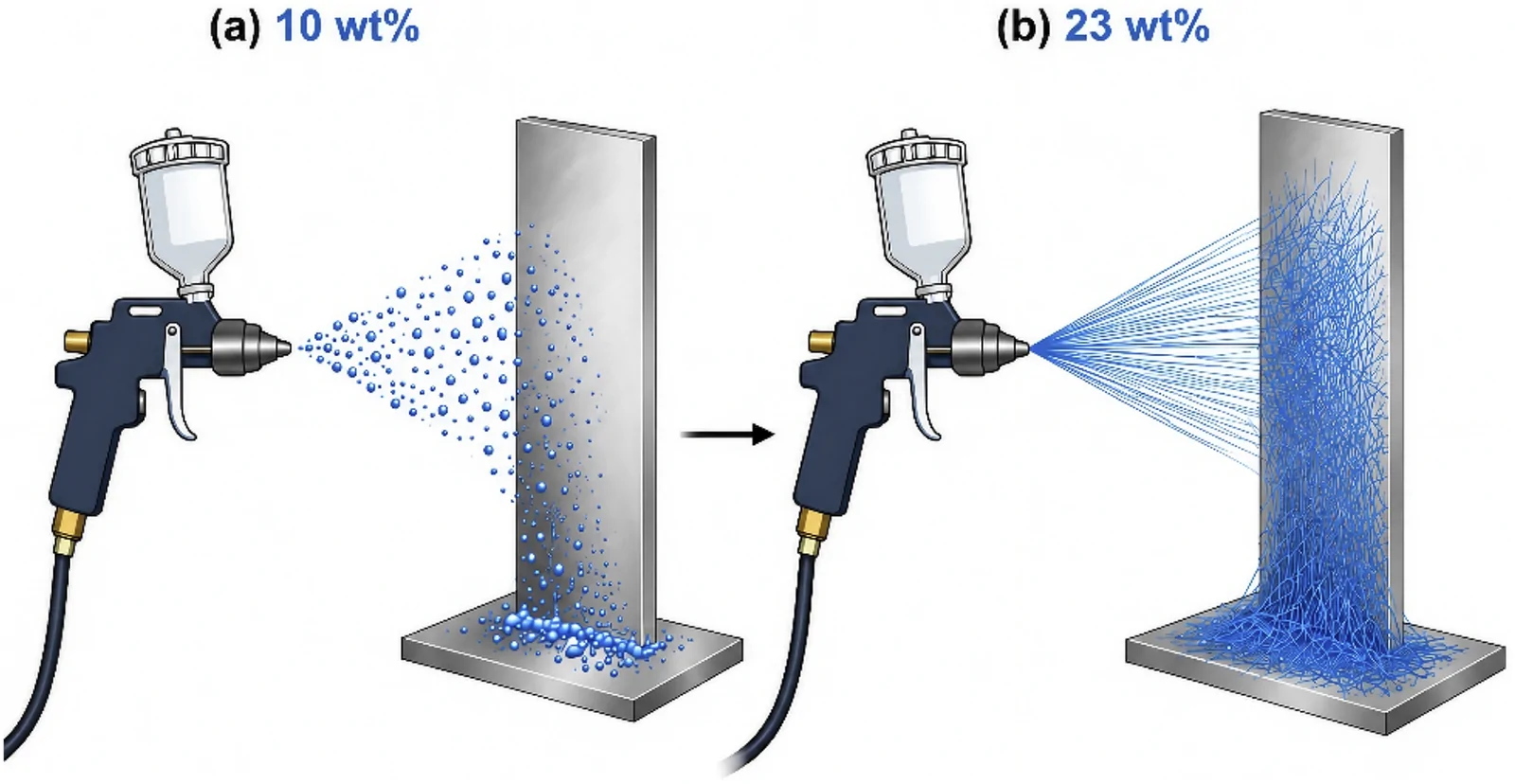
Representative deposition morphologies illustrating the concentration-dependent transition from droplet-dominated deposition at: (a) low polymer concentration (10 wt%) to (b) continuous fibre-forming deposition at high polymer concentration (23 wt%) during airbrush-based solution blow spinning.

Different working distances were used during formulation screening to account for concentration-dependent differences in spray behaviour. Lower viscosity formulations required longer spray distances to promote solvent evaporation and reduce wet droplet deposition, whereas higher viscosity formulations could be collected at shorter distances, improving fibre deposition and nonwoven mat formation.

Overall, fibre formation in the CA/PVAc system was governed by a narrow concentration-dependent processability window defined by two competing requirements: sufficient polymer chain entanglement to suppress capillary-driven jet breakup and sufficiently low solution viscosity to permit stable atomisation and spray delivery. Below this window, dilute solutions lacked adequate viscoelastic resistance and underwent droplet breakup, whereas above it, excessive viscosity impaired stable jet formation and nozzle flow. Stable nonwoven formation therefore emerged only within a high-concentration regime approaching or exceeding the fibre-forming threshold, where adequate chain entanglement, jet stability, and solvent evaporation collectively enabled continuous fibre deposition.^37,38^

### 3.2 Spray stability and deposition behaviour

Following identification of the 23 wt% fibre-forming formulation, spray stability was evaluated to assess process reproducibility under fixed operating conditions. Deposition behaviour was quantified gravimetrically by measuring the mass of material deposited on the collector at different spraying times Table 2.

**Table 2:** Gravimetric analysis of deposited mass and deposition rate of the CA/PVAc–acetone spray system at different spraying durations

Spray duration (s)	Deposited mass (mg)	Deposition rate (mg s^−1^)	Normalised deposition rate (mg cm^−2^ s^−1^)
5	70 ± 36	14.0 ± 7.2	0.56 ± 0.29
10	140 ± 20	14.0 ± 2.0	0.56 ± 0.08
15	230 ± 26	15.3 ± 1.8	0.61 ± 0.07

The deposited mass increased with spraying time, rising from 70 ± 36 mg after 5 s to 230 ± 26 mg after 15 s. More importantly, the normalised deposition rate remained relatively constant, ranging from 0.56 to 0.61 mg cm^−2^ s^−1^ once spraying was established, indicating stable material delivery under steady spraying conditions.

The higher variability observed at short spraying times (5 s) is considered to arise from the initial transient spraying regime, during which aerodynamic shear and solution flow had not yet fully stabilised. During this early stage, fluctuating jet behaviour may lead to variations in droplet breakup and deposition. This behaviour is consistent with mechanistic models of solution blow spinning (SBS), in which a stable jet is established only when the applied aerodynamic shear force exceeds droplet surface tension, enabling jet elongation and the transition from discontinuous jetting to continuous fibre formation.^39^

As spraying continued beyond approximately 10 s, the system reached a quasi-steady jetting regime, as evidenced by the relatively stable normalised deposition rate. Additional gravimetric measurements yielded an average solution flow rate of 38.3 ± 11.0 mg s^−1^. Although some variability is expected due to manual operation of the gravity-fed airbrush system, the observed stable deposition behaviour is consistent with previous SBS studies reporting steady jetting once continuous fibre formation is established under fixed operating conditions.^11,21^ The optimised CA/PVAc formulation consistently produced reproducible fibrous morphology and cohesive nonwoven mats, indicating that the system provided sufficiently stable and controllable deposition for reliable structural, thermal, and mechanical characterisation.

### 3.3 Morphological characterisation (optical microscopy and SEM)

Morphological characterisation was performed to visually validate the concentration-dependent fibre formation mechanism identified during formulation screening in Section 3.1. Representative intermediate formulations (17.2 and 18 wt%) were examined using scanning electron microscopy (SEM) to capture transitional morphological changes, while the low- and high-concentration benchmark formulations (10 and 23 wt%) were further analysed using both optical microscopy and SEM to compare the extreme non-fibre-forming and stable fibre-forming regimes.

Polarised optical microscopy further supported the concentration-dependent structural differences by revealing changes in surface coverage and layer continuity at the mesoscale Fig. 3. The 10 wt% sample exhibited irregular surface coverage characterised by isolated droplets and polymer agglomeration. Such behaviour is commonly observed in low-solids spray systems, where limited polymer content prevents the formation of a continuous polymer network, while evaporation-induced capillary flow and contact-line pinning promote outward solute transport and peripheral material accumulation during drying, resulting in characteristic ring-like deposits (coffee-ring effect).,^40^ whereas the 23 wt% formulation showed a more uniform multilayer fibrous structure with improved surface continuity, indicating successful fibre accumulation and overlap during repeated spray deposition.

**Fig. 3:**
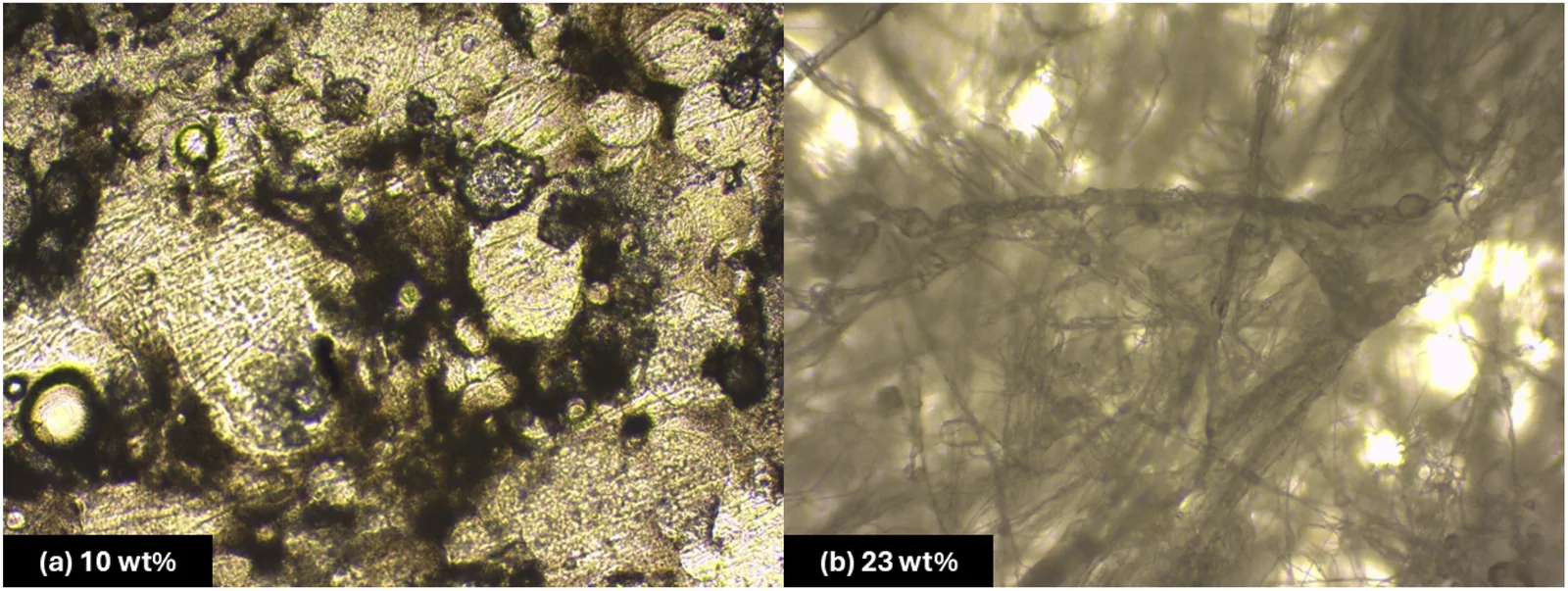
Optical microscopy images of CA/PVAc sprayed mats demonstrating concentration-dependent morphological evolution: (a) 10 wt% showing droplet-dominated deposition with polymer agglomeration; (b) 23 wt% exhibiting interconnected fibre formation with reduced droplet deposition.

SEM observations further confirmed a clear concentration-dependent morphological transition in the deposited CA/PVAc structures Fig. 4. At the lowest polymer concentration (10 wt%), the deposited material consisted predominantly of droplets and agglomerated particulate features, with no evidence of continuous fibre formation, indicating extensive jet breakup during spraying. Increasing the total polymer concentration to 17.2 wt% resulted in the emergence of discontinuous fibre segments accompanied by bead-like structures, suggesting partial jet stabilisation but insufficient continuity for coherent fibre network formation. At 18 wt%, fibre formation became more pronounced, with reduced droplet content and improved fibre continuity, although residual bead defects remained visible. In contrast, the optimised 23 wt% formulation exhibited a continuous interconnected fibrous network with minimal bead formation, indicating successful establishment of a stable fibre-forming spray regime.

**Fig. 4:**
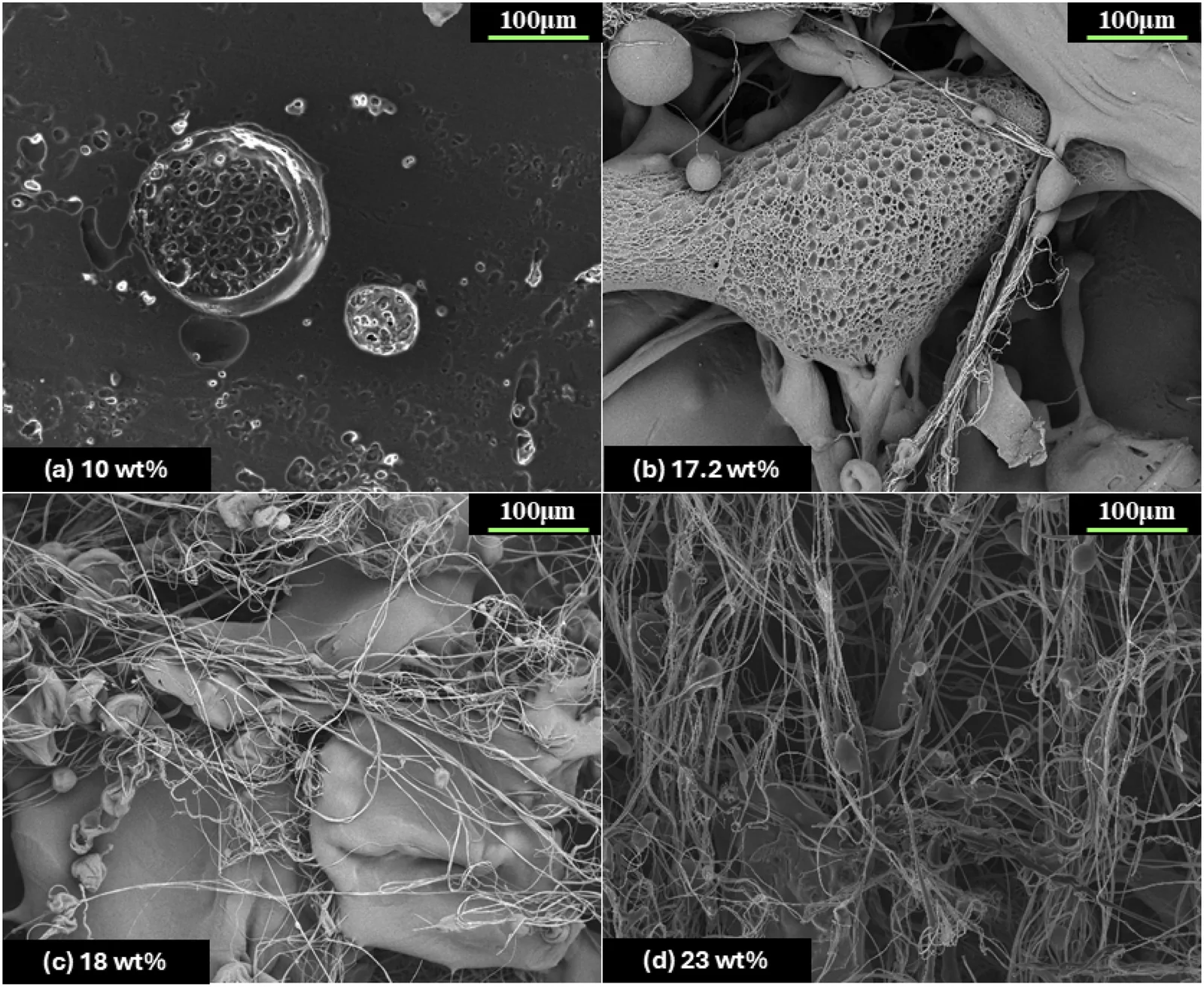
SEM micrographs of CA/PVAc sprayed mats at different polymer concentrations showing concentration-dependent morphological evolution from droplet-dominated deposition to continuous fibre formation.

High-magnification SEM further confirmed the absence of continuous fibres in the 10 wt% sample, whereas detailed analysis of the optimised 23 wt% formulation revealed an interconnected fibrous network with predominantly random fibre orientation and multiple fibre–fibre junctions, characteristic of nonwoven structures formed through turbulent spray deposition Fig. 5. Although occasional bead-like defects remained visible, continuous fibre formation was maintained throughout the deposited network, indicating effective suppression of extensive jet breakup under the selected processing conditions.

**Fig. 5:**
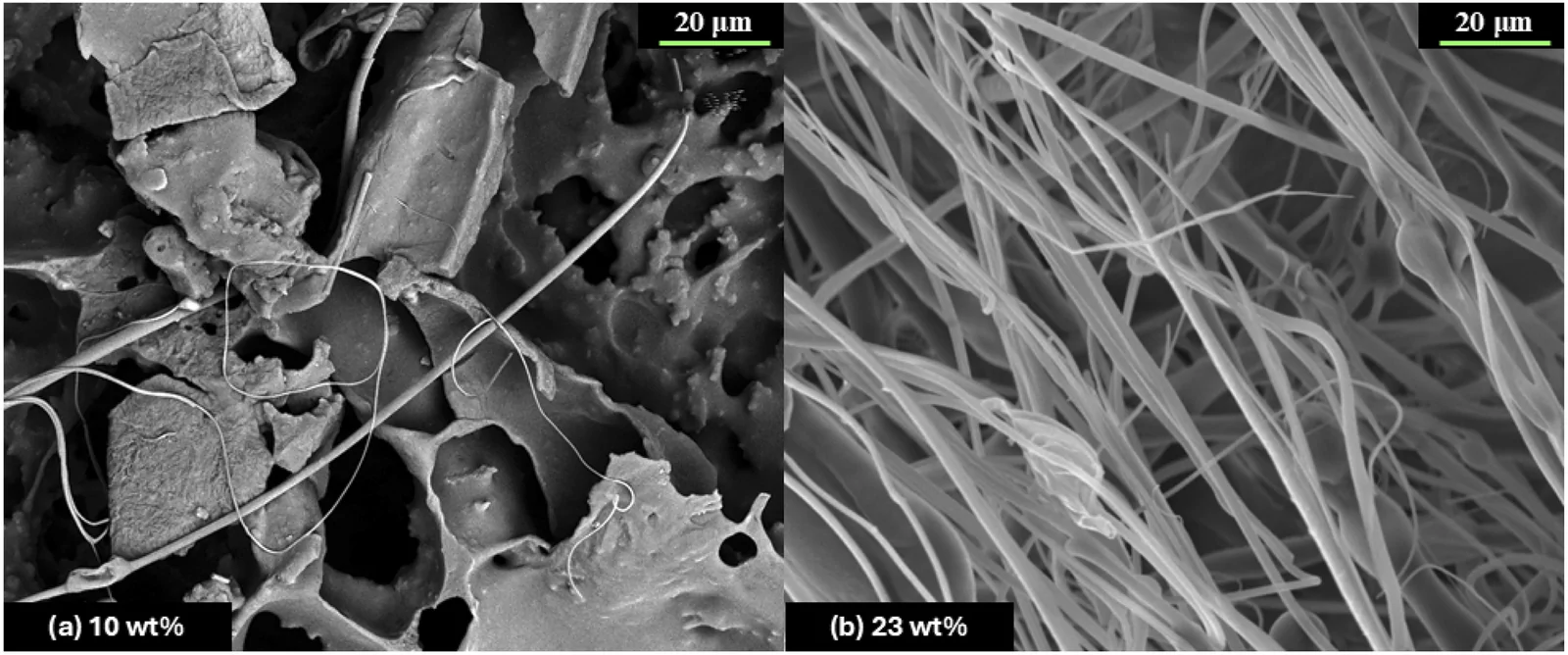
Higher-magnification SEM micrographs (scale bar: 20 µm) showing microstructural differences between CA/PVAc sprayed mats prepared at (a) 10 wt% and (b) 23 wt%.

Quantitative fibre diameter analysis was performed only for the optimised 23 wt% formulation, as continuous fibres were not formed in the 10 wt% sample and therefore reliable diameter measurement was not possible.

A total of 100 individual fibres were measured from representative SEM micrographs using ImageJ. The fibre diameter histogram Fig. 6 showed that most fibres were distributed within the submicron-to-low-micron range, with moderate diameter variability. The statistical summary presented in Table 3 yielded an average fibre diameter of 0.97 ± 0.41 µm, with a measured range of 0.37–2.25 µm, indicating the formation of submicron-to-micron scale fibres.

**Fig. 6:**
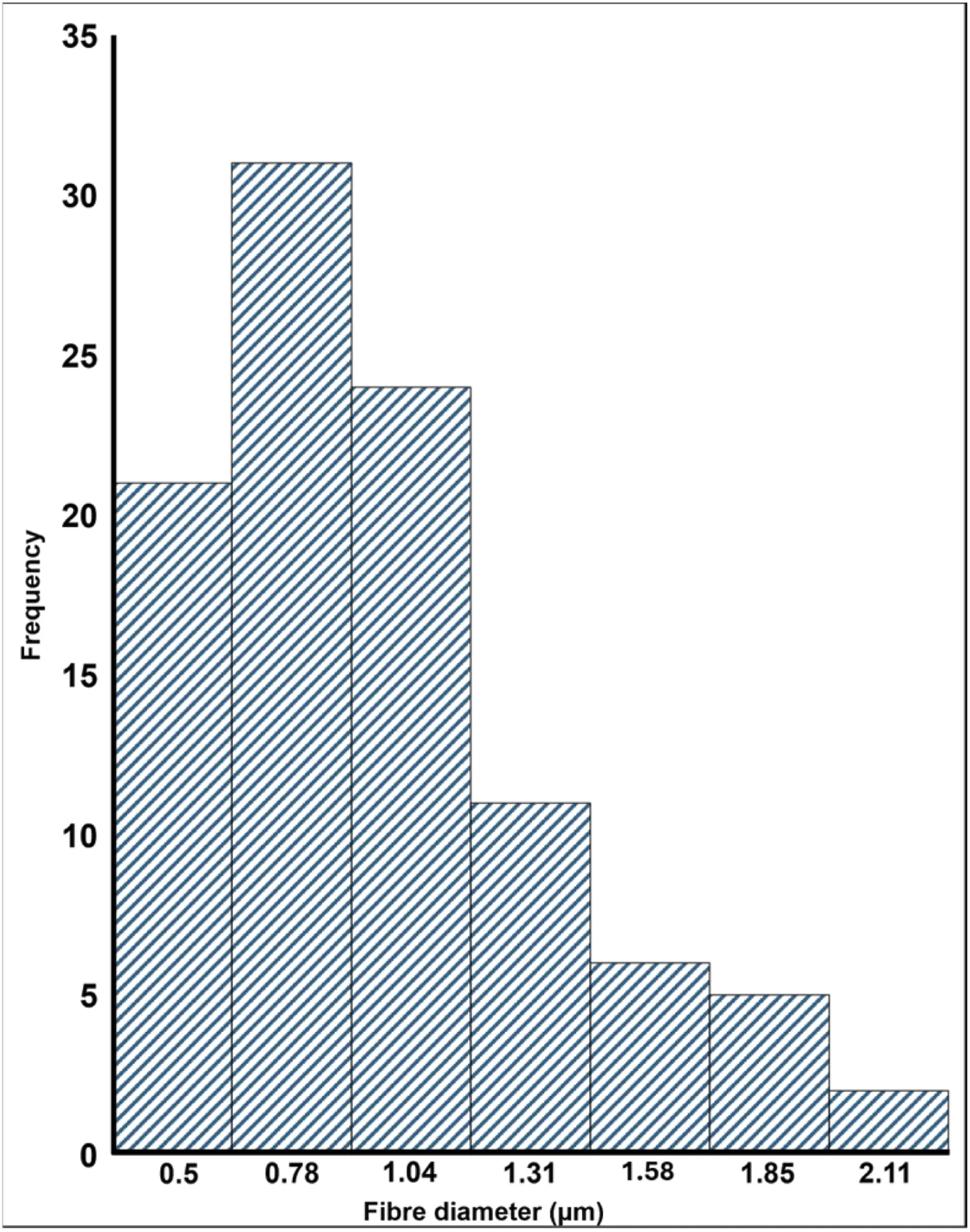
Fibre diameter distribution of the optimised 23 wt% CA/PVAc nonwoven mat measured from SEM micrographs (*n* = 100).

**Table 3:** Statistical summary of fibre diameter measurements for the optimised 23 wt% CA/PVAc nonwoven mat obtained from SEM analysis

Parameter	Value
Number of measured fibres, *n*	100
Average fibre diameter (µm)	0.97
Standard deviation (µm)	0.41
Diameter range (µm)	0.37–2.25
Coefficient of variation (%)	42.5

The observed distribution reflects moderate variability in fibre size, which is expected in airbrush-based SBS due to local fluctuations in jet stretching, aerodynamic shear, and solvent evaporation during fibre formation.

### 3.4 Chemical and thermal characterisation (FTIR/TGA and DSC)

#### 3.4.1 FTIR analysis of chemical integrity and intermolecular interactions

FTIR spectra of neat cellulose acetate (CA), neat poly(vinyl acetate) (PVAc), and the sprayed CA/PVAc nonwoven mat are shown in Fig. 7 to evaluate the chemical structure and assess the preservation of functional groups after processing.

**Fig. 7:**
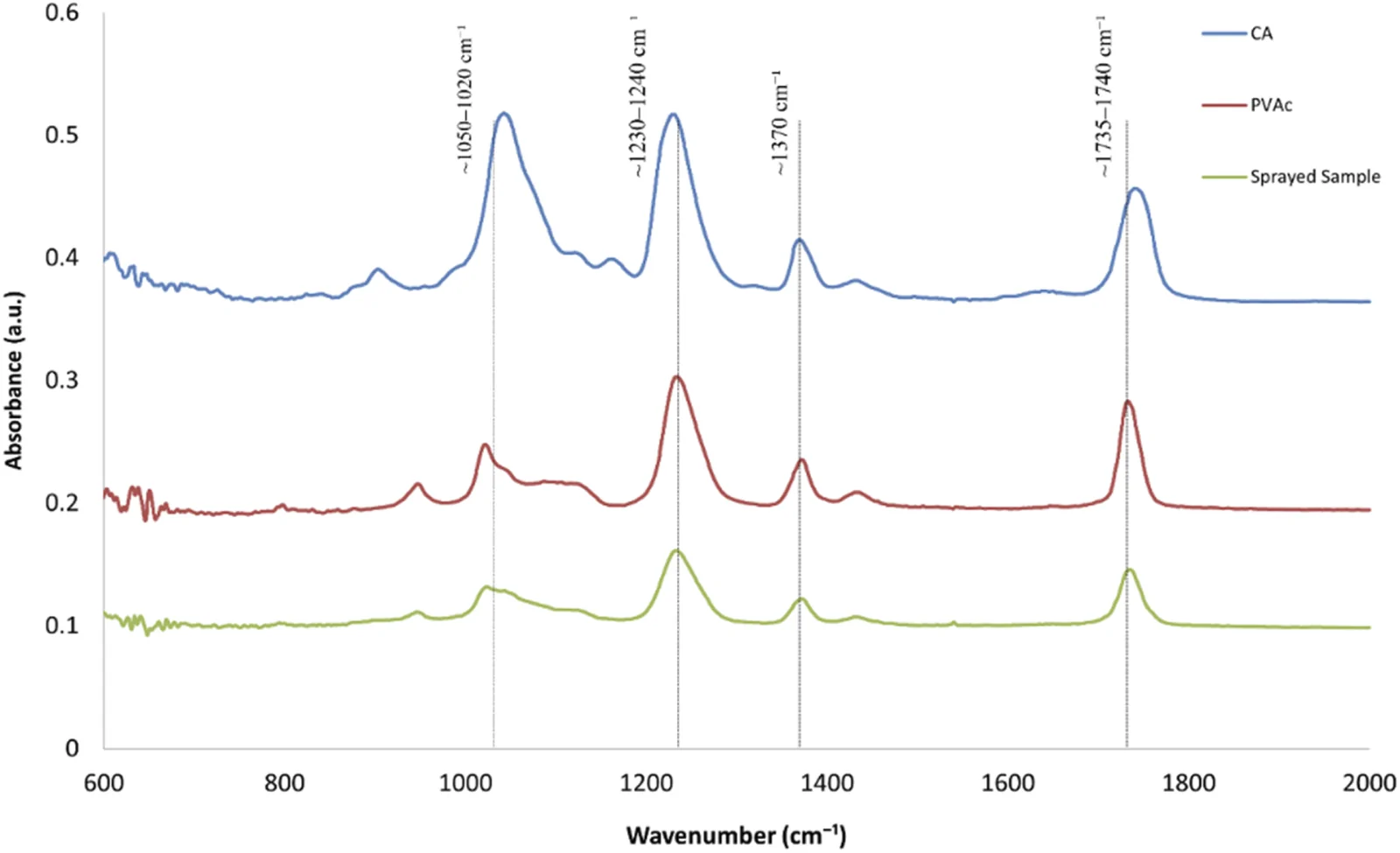
FTIR spectra of neat cellulose acetate (CA), neat poly(vinyl acetate) (PVAc), and the sprayed CA/PVAc nonwoven mat showing preservation of characteristic functional groups after airbrush-based solution blow spinning.

Neat CA exhibited characteristic absorption bands at ∼1735–1740 cm^−1^ corresponding to ester carbonyl (C=O) stretching vibrations, together with bands at ∼1370 cm^−1^ associated with C–H bending vibrations. Additional strong absorption bands were observed at ∼1230–1240 cm^−1^ and ∼1050 cm^−1^, corresponding to C–O–C and C–O stretching vibrations, respectively, consistent with reported literature values for acetylated cellulose derivatives.^29^

Neat PVAc displays a strong acetate ester absorbance band at ∼1732 cm^−1^, together with C–O stretching bands at ∼1230 and ∼1020 cm^−1^.^41^ An additional band at ∼1370 cm^−1^ is attributed to CH_3_ bending vibrations, consistent with the aliphatic backbone of PVAc.^42^

The FTIR spectrum of the sprayed CA/PVAc nonwoven mat showed the presence of characteristic absorption bands from both CA and PVAc, confirming preservation of the chemical structures of both polymer components after spray processing. The carbonyl band observed at ∼1735 cm^−1^ represents the overlapping C=O stretching vibrations of CA and PVAc, while the C–O and C–O–C stretching region remained visible, with peaks at ∼1233 cm^−1^ and ∼1023 cm^−1^, while CH_3_ bending vibrations appeared at ∼1370 cm^−1^. No new absorption peaks or significant disappearance of functional-group signals were observed, indicating that the solution blow spinning process did not induce chemical degradation or formation of new covalent bonds.

A slight spectral shift of approximately ∼5 cm^−1^ in the carbonyl region suggests weak intermolecular interactions between CA and PVAc, potentially arising from secondary interactions such as hydrogen bonding. Similar minor spectral perturbations have been reported in cellulose acetate-based systems while retaining the fundamental chemical structure of the constituent polymers after processing.^25^

Overall, the FTIR results confirm that airbrush-based solution blow spinning preserved the chemical integrity of the CA/PVAc formulation, indicating that fibre formation and nonwoven mat development occurred through physical jet stretching, solvent evaporation, and polymer deposition rather than chemical crosslinking or covalent bond formation.

#### 3.4.2 Thermogravimetric analysis (TGA)

Thermogravimetric analysis (TGA) was performed to evaluate the thermal stability and degradation behaviour of the sprayed CA/PVAc nonwoven material. The TGA curves of neat cellulose acetate (CA), neat poly(vinyl acetate) (PVAc), and the sprayed CA/PVAc nonwoven mat are shown in Fig. 8.

**Fig. 8:**
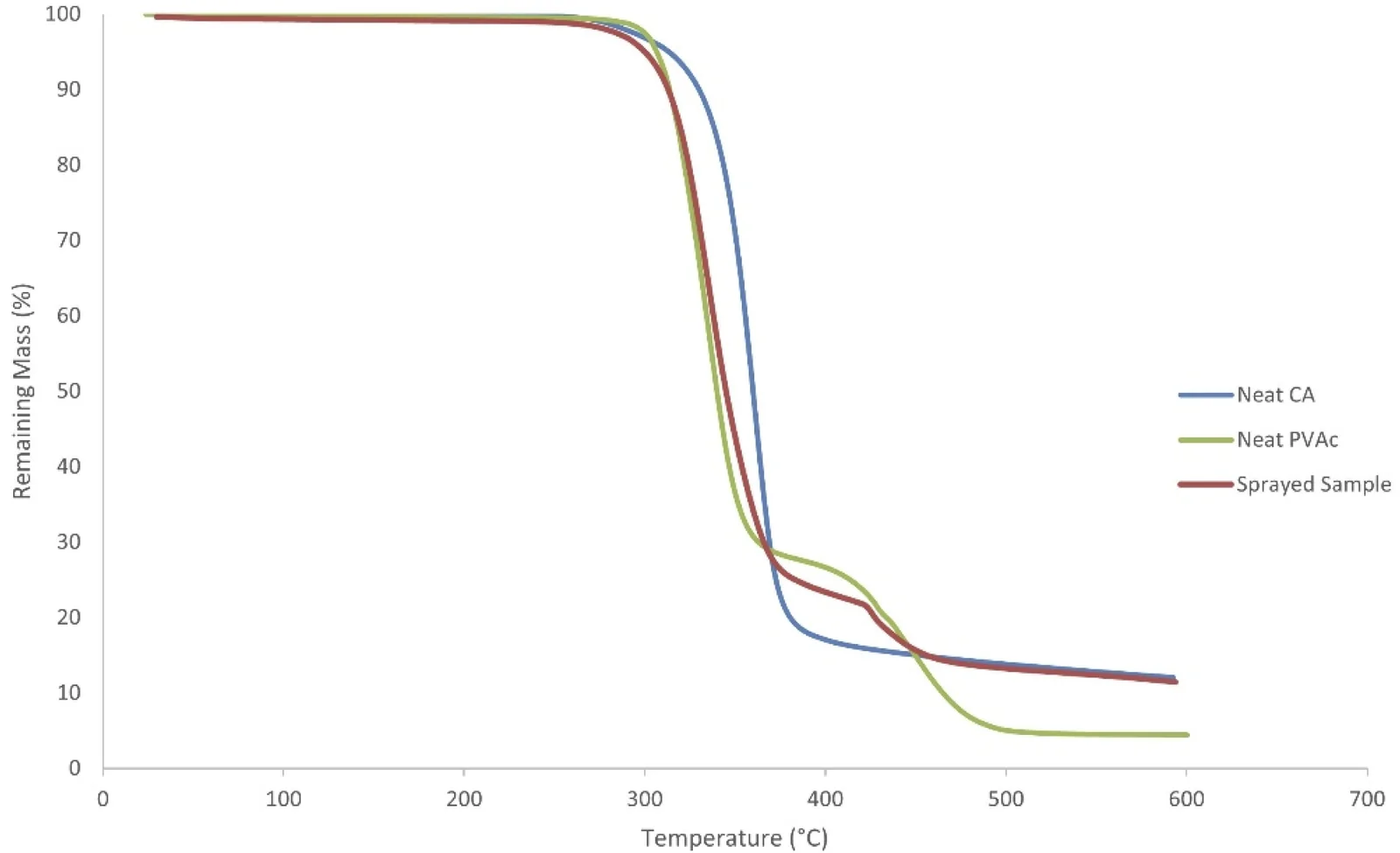
TGA curves of neat cellulose acetate (CA), neat poly(vinyl acetate) (PVAc), and the sprayed CA/PVAc nonwoven mat.

Neat cellulose acetate exhibited two thermal events. A small initial weight loss of approximately 1% below 120 °C was observed, corresponding to the evaporation of absorbed moisture. The main degradation step occurred as a single dominant event, with *T*_onset_ = 341.34 °C and *T*_end_ = 374.17 °C, corresponding to 87.72% mass loss. At higher temperatures, the residual mass decreased gradually, leaving approximately 16% char at 600 °C.

Neat PVAc exhibited a two-step thermal degradation behaviour. The first and most significant degradation stage occurred between 320 and 355 °C, with a mass loss of approximately 43.64%, and a DTG maximum at approximately 333 °C. The second degradation stage appeared between 400 and 470 °C, corresponding to an additional 13.50% mass loss, with a DTG maximum at approximately 455 °C. Upon heating to 600 °C, a small residual mass remained.

The sprayed CA/PVAc nonwoven mat exhibited overlapping degradation behaviour characteristic of both polymer components, with two main mass-loss stages. The first degradation event occurred between *T*_onset1_ = 316.46 °C and *T*_end1_ = 358.68 °C, corresponding to 73.76% mass loss, with a DTG maximum at *T*_max1_ = 337.78 °C. A second degradation stage was observed between *T*_onset2_ = 423.98 °C and *T*_end2_ = 445.99 °C, corresponding to 12.41% mass loss, with a DTG maximum at approximately 445 °C. The blended sample exhibited a residual mass of approximately 21% at 600 °C.

The thermal degradation behaviour of cellulose acetate is primarily associated with decomposition of acetate functional groups followed by scission of the glucopyranose backbone, typically producing a single dominant degradation step and measurable char residue.^43^ In contrast, PVAc commonly undergoes two-stage degradation, where the first stage corresponds to deacetylation and elimination of acetic acid, generating unsaturated polyene sequences, followed by backbone degradation at higher temperatures.^41^ The degradation profile of the sprayed CA/PVAc nonwoven reflects the combined contribution of these two polymers.

Notably, the first degradation stage of the blended sample occurred at a lower onset temperature than neat CA but remained close to the primary degradation region of PVAc. This suggests that the initial thermal decomposition was dominated by overlapping PVAc deacetylation and CA acetate-group decomposition. The second degradation stage near 445 °C is attributed to further degradation of the polyene/backbone formed after PVAc deacetylation together with the decomposition of char residues generated during CA degradation.^44,45^

Overall, the TGA results indicate that airbrush-based solution blow spinning did not substantially alter the intrinsic thermal degradation behaviour of either polymer. The blended nonwoven retained thermal stability well above ambient and typical textile processing temperatures, indicating that fibre formation occurred through physical jet stretching and solvent evaporation rather than thermally induced chemical transformation.

#### 3.4.3 Differential scanning calorimetry (DSC)

Differential Scanning Calorimetry (DSC) was performed to evaluate the thermal behaviour of neat cellulose acetate (CA), neat poly(vinyl acetate) (PVAc), and the sprayed CA/PVAc nonwoven mat. The corresponding thermograms are shown in Fig. 9–11.

**Fig. 9:**
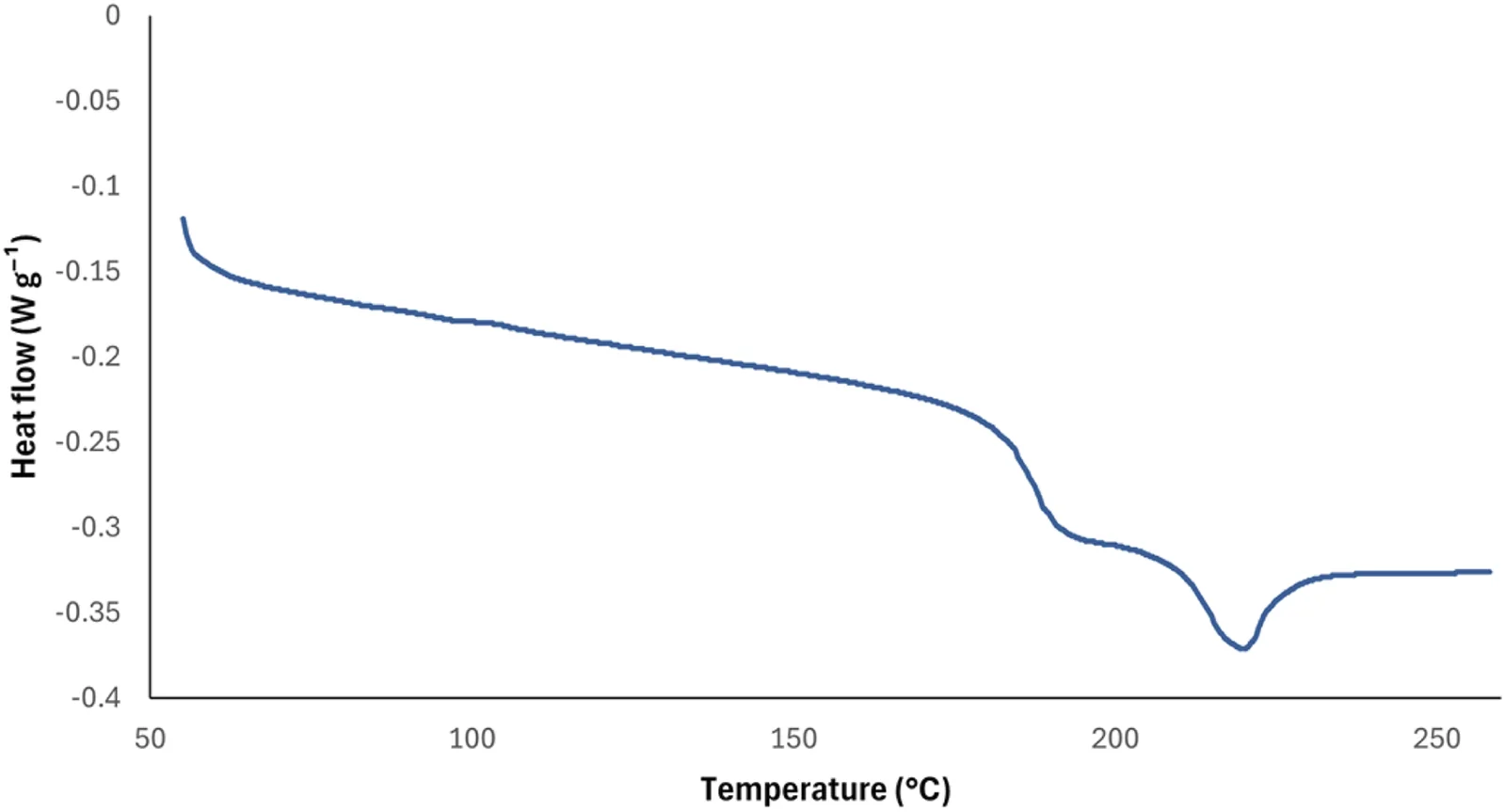
DSC thermogram of neat cellulose acetate (CA) showing a broad glass transition (*T*_g_ = 184.9 °C) and absence of melting or crystallisation peaks, consistent with its predominantly amorphous structure.

**Fig. 10:**
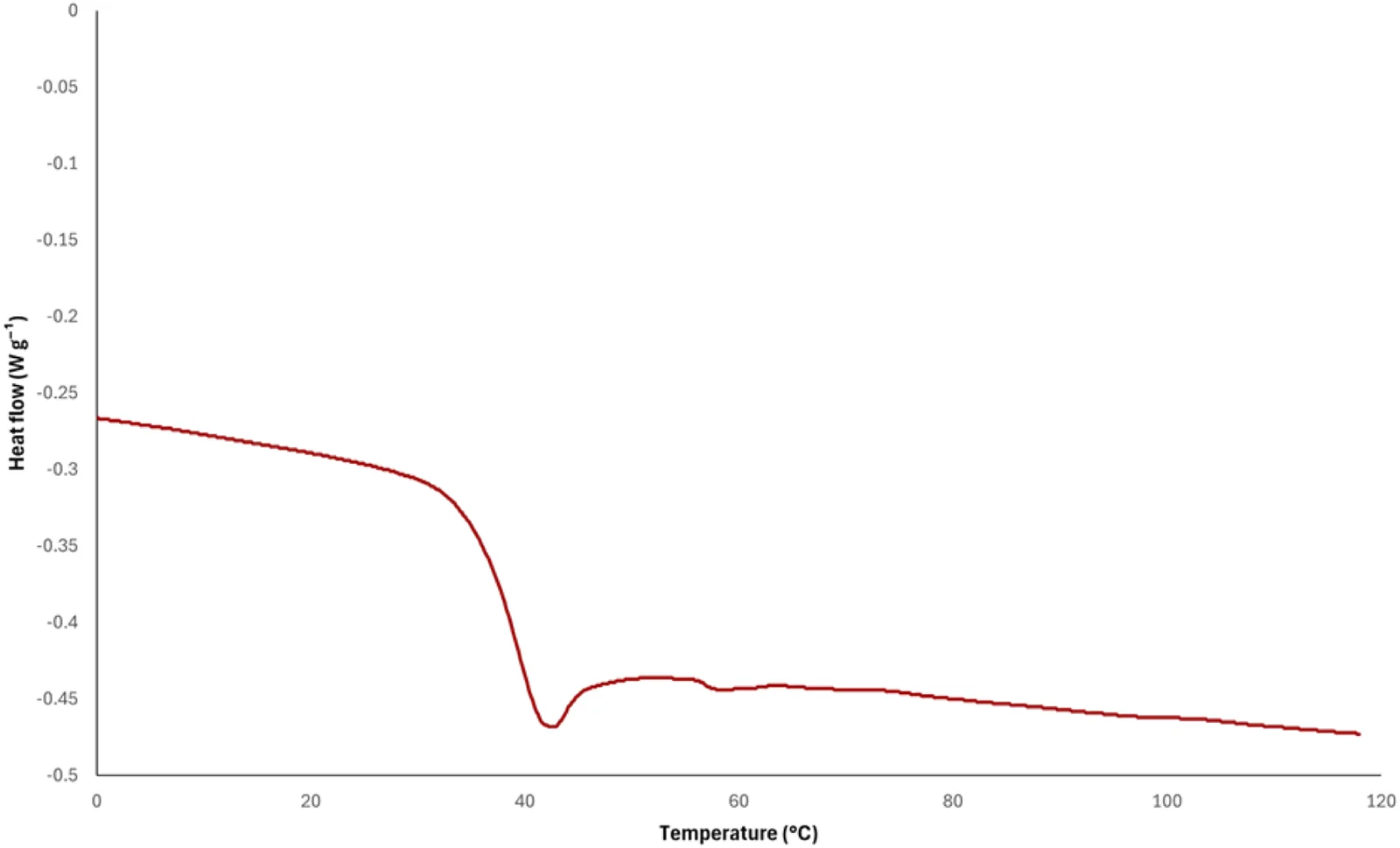
DSC thermogram of neat poly(vinyl acetate) (PVAc) showing a glass transition (*T*_g_ ≈ 38 °C) and no observable melting or crystallisation peaks within the scanned temperature range, consistent with its amorphous nature.

**Fig. 11:**
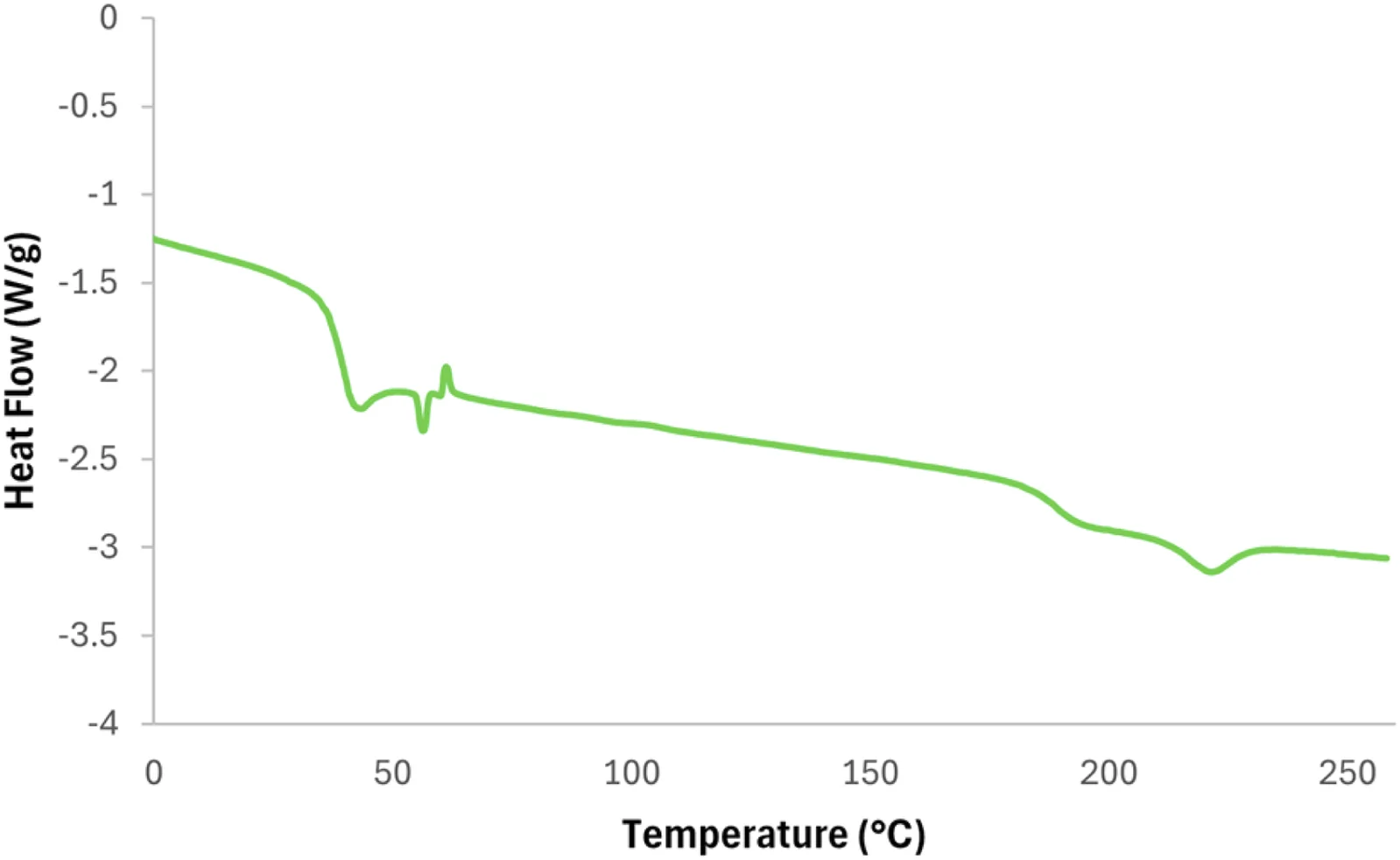
DSC thermogram of the sprayed CA/PVAc nonwoven mat exhibiting a single glass transition (*T*_g_ ≈ 38 °C) with no observable melting peak, suggesting dominance of the PVAc-rich amorphous phase.

The DSC thermogram of neat cellulose acetate powder in Fig. 9 exhibited a broad glass-transition region appearing as a gradual baseline shift rather than a sharp step transition. Using the midpoint (half-height) method, the glass-transition temperature (*T*_g_) was determined to be approximately 184.9 °C. No distinct melting or crystallisation peaks were observed within the scanned temperature range up to 260 °C.

The broad glass-transition behaviour of CA reflects its predominantly amorphous structure and restricted chain mobility. The absence of melting or crystallisation peaks further indicates that CA does not exhibit a distinct melting transition under the present experimental conditions but instead undergoes thermal softening prior to thermal degradation, consistent with the reported thermal behaviour of cellulose acetate.^29^

For neat PVAc Fig. 10, a well-defined glass transition was observed near ∼38 °C, consistent with the reported *T*_g_ range of PVAc (∼38–50 °C). No melting or crystallisation peaks were detected within the measured temperature range.

The absence of melting behaviour indicates that PVAc is also predominantly amorphous. As reported in the literature, PVAc does not display a true melting transition but instead undergoes thermal degradation at higher temperatures, beyond the DSC measurement window.^41^

The sprayed CA/PVAc nonwoven mat Fig. 11 exhibited predominantly amorphous thermal behaviour, with no detectable melting or crystallisation peaks. A single glass-transition event was observed as a step change in the heat-flow baseline within the temperature range of approximately 20–60 °C, with a *T*_g_ of ∼38 °C, closely matching that of neat PVAc.

The presence of a single detectable glass transition suggests that the thermal response of the blended system is dominated by the PVAc-rich amorphous phase, while the glass transition associated with CA was not distinctly resolved. This may be attributed to the inherently broad and weak thermal signal of CA, combined with its lower mobility and reduced sensitivity in the blend. The absence of a clearly resolved second *T*_g_ suggests that the thermal contribution of the CA-rich phase was either weak, broadly distributed, or overlapped with the dominant amorphous response of PVAc, preventing clear resolution under the present measurement conditions.

The absence of a clearly resolved second *T*_g_ is consistent with DSC observations reported for partially miscible polymer blends, in which overlapping glass-transition regions or a single broad transition may arise because of limited phase separation and close proximity of the component *T*_g_ values.^46^

Overall, the DSC results indicate that the sprayed CA/PVAc nonwoven retained a predominantly amorphous structure after deposition. The absence of melting transitions and the dominance of a single PVAc-associated glass transition suggest limited structural ordering and thermal behaviour largely governed by the PVAc-rich amorphous phase.

### 3.5 Physical and mechanical properties (thickness/porosity/tensile)

#### 3.5.1 Thickness and porosity

The sprayed CA/PVAc nonwoven mat exhibited an average thickness of 0.595 ± 0.049 mm (*n* = 5), with individual values ranging from 0.548 to 0.675 mm, as summarised in Table 4. In addition, the material exhibited an average apparent density of 0.415 ± 0.043 g cm^−3^ and an average porosity of 66.0 ± 3.5%, indicating the formation of a highly porous fibrous structure.

**Table 4:** Thickness, apparent density, and porosity of sprayed CA/PVAc nonwoven samples (*n* = 5)

Sample	Average thickness (mm)	Mass (g)	Apparent density (g cm^−3^)	Porosity (%)
S1	0.675	0.0240	0.356	70.8
S2	0.609	0.0242	0.397	67.5
S3	0.582	0.0235	0.419	65.7
S4	0.548	0.0250	0.430	64.8
S5	0.561	0.0259	0.473	61.2
**Mean** ± **SD**	**0.595 ± 0.049**	**0.0245 ± 0.001**	**0.415 ± 0.043**	**66.0 ± 3.5**

The measured thickness reflects progressive fibre accumulation during repeated airbrush-based solution blow spinning deposition. The multilayer spraying strategy, incorporating intermediate drying intervals between successive layers, facilitated sequential material build-up and promoted the formation of a self-supporting three-dimensional nonwoven architecture. Unlike dense cast polymer films, the deposited material retained substantial internal void volume despite reaching a thickness of approximately 0.6 mm.

SEM observations further supported this interpretation, revealing a randomly oriented fibrous network containing visible interfibre void regions. These morphological features are consistent with the gravimetrically determined porosity values and confirm that the deposited structure consists of an interconnected nonwoven network rather than a compact continuous film.

The substantial porosity observed in the present system can be attributed to the characteristic deposition mechanism of solution blow spinning, in which aerodynamic stretching of the polymer jet and rapid solvent evaporation promote fibre solidification prior to collector impact.^47^ This process facilitates the formation of loosely packed fibrous networks with significant void spaces between deposited fibres. Similar porous architectures have been reported in solution blow spun systems and polyurethane-based fibrous scaffolds.^48,49^

The observed porosity of the CA/PVAc system may also reflect partial fibre packing and local fibre–fibre adhesion during multilayer deposition. The presence of PVAc likely contributed to interfibre bonding, improving structural cohesion while partially reducing the overall void fraction compared with highly open nanofibrous mats. This balance between fibre connectivity and internal porosity is particularly important for textile-relevant nonwoven materials, where sufficient structural integrity must be achieved without sacrificing flexibility and air-filled architecture.

#### 3.5.2 Tensile properties

The tensile properties of the CA/PVAc nonwoven are summarized in Table 5, and representative stress–strain curves for five replicate specimens are shown in Fig. 12.

**Table 5:** Mechanical properties of the optimised sprayed CA/PVAc nonwoven (*n* = 5)

Property	Mean ± SD
Max force (N)	0.409 ± 0.148
Tensile strength (MPa)	0.052 ± 0.017
Elongation at break (%)	18.0 ± 7.7
Apparent Young's modulus (MPa)	1.53 ± 0.48

**Fig. 12:**
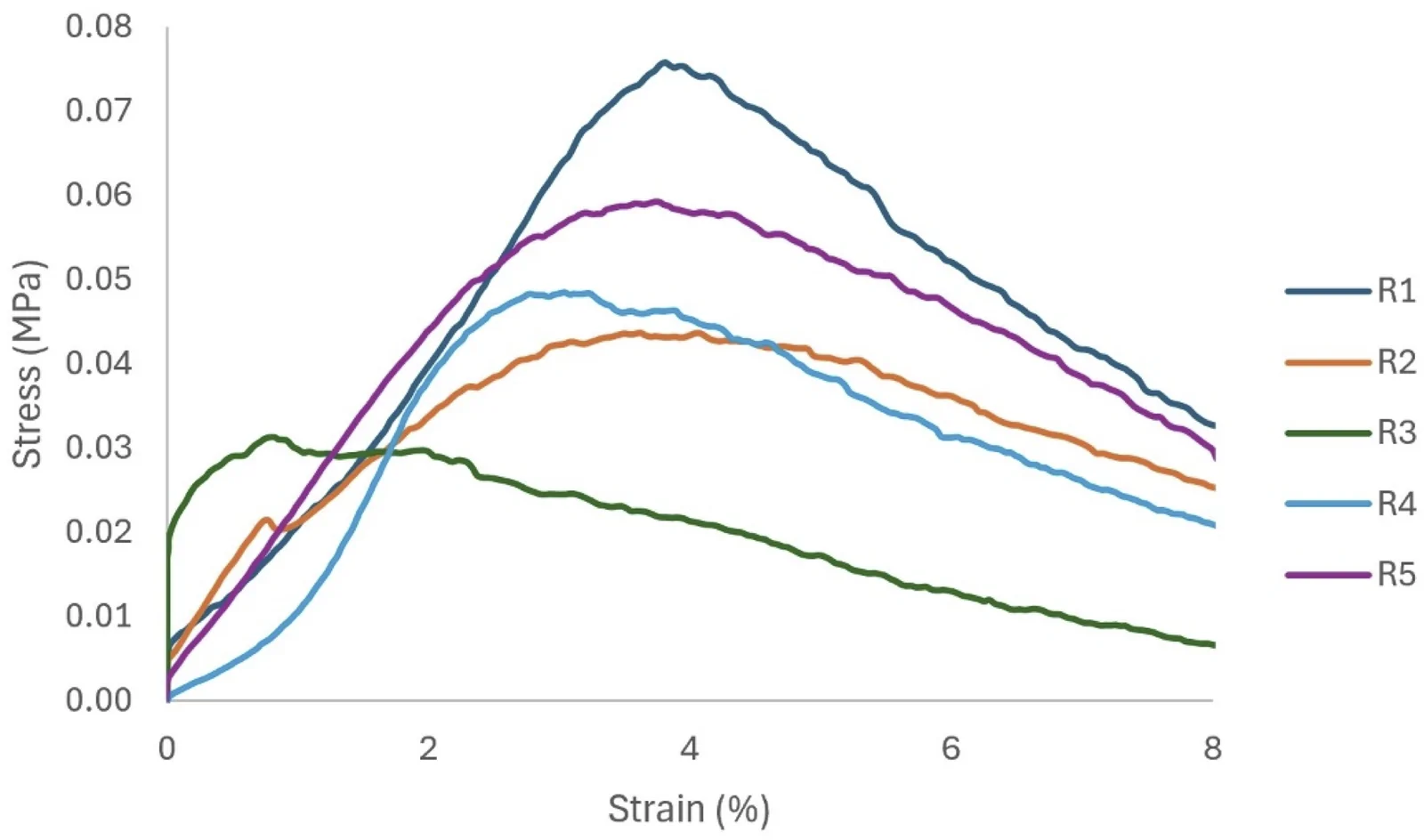
Representative stress–strain curves of five replicate sprayed CA/PVAc nonwoven specimens.

The material exhibited an average maximum force of 0.409 ± 0.148 N, corresponding to a tensile strength of 0.052 ± 0.017 MPa. The average elongation at break was 18.0 ± 7.7%, while the apparent Young's modulus was 1.53 ± 0.48 MPa (*n* = 5).

All specimens exhibited a similar stress–strain response, characterised by an initial increase in stress to a maximum value followed by a gradual decrease. The absence of abrupt fracture suggests progressive failure of the fibrous network during tensile loading rather than catastrophic brittle rupture. Such behaviour is consistent with porous nonwoven structures, where load transfer occurs through interconnected fibres and bonded fibre junctions rather than through a continuous polymer matrix. SEM analysis revealed a predominantly fibrous architecture with occasional fused fibre junctions, supporting this deformation mechanism.^50^

The relatively low tensile strength is consistent with the highly porous structure of the material. As shown in Section 3.5.1, the sprayed nonwoven exhibited an average porosity of 66.0 ± 3.5%, indicating that a substantial proportion of the sample volume consisted of void spaces rather than load-bearing polymer. Consequently, only a fraction of the cross-sectional area contributed directly to stress transfer, resulting in reduced load-bearing capacity and a relatively low apparent modulus.

The measured thickness further supports the formation of a lightweight self-supporting fibrous network rather than a dense polymer film, helping to explain how structural cohesion was maintained despite the high internal porosity.

Despite the highly porous architecture, the material retained moderate extensibility, reaching an average elongation at break of 18.0 ± 7.7%. Similar strain-at-failure behaviour has been reported for solution blow spun PA6 nanofibre mats, which exhibited approximately 20% strain before rupture.^50^ The observed extensibility may be attributed to the fibrous architecture of the network, which allows local fibre rearrangement and redistribution of stress during tensile deformation.^51^

Variation between replicate specimens was expected because the samples were produced by manual airbrush deposition, where small differences in local fibre packing, bonded junction density, thickness, and pore distribution may influence stress transfer. Nevertheless, the overall similarity of the stress–strain profiles indicates that the optimised formulation produced a mechanically coherent self-supporting nonwoven structure suitable for further mechanical optimisation.

## 4 Limitations and future perspectives

While the present study demonstrates the feasibility of producing cohesive spray-formed nonwoven structures *via* airbrush-based solution blow spinning, several limitations should be acknowledged. The current work focused on establishing the concentration-dependent fibre formation window and characterising the resulting morphology, thermal behaviour, and preliminary mechanical performance. Although fibre formation was qualitatively correlated with polymer concentration, detailed rheological analysis to quantify the critical entanglement concentration and solution viscoelastic behaviour was beyond the scope of this study. In addition, key processing parameters including humidity, aerodynamic spray conditions (such as airflow and operating pressure), nozzle geometry, and spray distance were not systematically investigated, despite their known influence on jet stability, fibre morphology, and pore structure. Mechanical evaluation was also limited to preliminary tensile testing, while textile-relevant functional properties such as softness, drape, and breathability were not quantitatively assessed.

Future work will focus on establishing quantitative structure–property relationships through rheological characterisation and expanded mechanical evaluation of the resulting nonwoven materials, while further investigating how aerodynamic spray conditions and other processing parameters influence jet stability, fibre morphology, porosity, and deposition uniformity. In line with sustainability objectives, future studies will also explore fully bio-based binder systems, greener solvent alternatives, and scale-up of the airbrush-based process for controlled large-area textile fabrication.

## 5 Conclusions

This study demonstrated the successful fabrication of cohesive nonwoven structures from cellulose acetate (CA) and poly(vinyl acetate) (PVAc) using an airbrush-based solution blow spinning (SBS) process. An epoxy-free CA/PVAc formulation was developed to produce peelable, textile-like spray-formed mats without the need for additional crosslinking or post-processing steps.

Polymer concentration was identified as the critical parameter governing fibre formation and nonwoven cohesion. A clear concentration-dependent transition from droplet-dominated deposition to continuous fibre formation was observed, with stable fibrous mat formation achieved at 23 wt% total polymer concentration. This transition was governed by the balance between capillary-driven jet breakup, polymer chain entanglement, solution viscoelasticity, and aerodynamic stretching during spray deposition. Below the fibre-forming threshold, insufficient chain entanglement resulted in droplet breakup, whereas excessive viscosity impaired sprayability and nozzle flow.

Morphological analysis confirmed the formation of a continuous interconnected fibrous network with a mean fibre diameter of 0.97 ± 0.41 µm. The resulting nonwoven exhibited an average thickness of 0.595 ± 0.049 mm and a porosity of 66.0 ± 3.5%, indicating a lightweight porous architecture. FTIR analysis confirmed preservation of the chemical integrity of both polymer components, while TGA and DSC demonstrated adequate thermal stability and predominantly amorphous behaviour of the sprayed structure. Preliminary tensile testing further confirmed that the optimised formulation produced a mechanically coherent self-supporting nonwoven structure.

Overall, this work provides mechanistic insight into spray-based fibre formation and demonstrates that airbrush-based SBS offers a simple, low-cost, and scalable route for producing cohesive nonwoven structures from biodegradable polymer systems. These findings expand the application potential of solution blow spinning beyond conventional biomedical and filtration applications toward functional spray-deposited fibrous materials requiring lightweight, conformable, and removable fibrous layers, including disposable textile materials, protective coverings, and other on-demand spray-deposited nonwoven systems.

## Author contributions

Maria Emad Bashawri: conceptualization, methodology, investigation, formal analysis, data curation, visualization, project administration, writing – original draft, writing – review and editing. Abimbola O. Orisawayi: review & editing. Sameer S. Rahatekar, and Krzysztof K. Koziol: conceptualization, supervision, visualisation, writing – review and editing, and resources.

## Conflicts of interest

The authors declare that they have no known competing financial interests or personal relationships that could have appeared to influence the work reported in this paper.

## Data Availability

The data supporting the findings of this study are available from the corresponding author upon reasonable request.
